# Efficacy of Combination Therapy with Epinephrine Local Injection and Hemostatic Clips on Active Diverticular Bleeding

**DOI:** 10.3390/jcm11175195

**Published:** 2022-09-02

**Authors:** Seiji Hamada, Akira Teramoto, Ryuta Zukeyama, Shinobu Matsukawa, Tomofumi Fukuhara, Ryo Takaki, Takahiro Utsumi, Masamoto Nakamura, Kasen Kobashikawa, Nobufumi Uchima, Tomokuni Nakayoshi, Fukunori Kinjo

**Affiliations:** 1 Gastrointestinal Center, Urasoe General Hospital, Urasoe 901-2132, Japan; 2 Third Department of Internal Medicine, University of Toyama, Toyama 930-0194, Japan; 3 Department of Gastroenterology and Hepatology, Kyoto University Graduate School of Medicine, Kyoto 606-8507, Japan; 4 Department of Gastroenterology, Shonan Hospital, Okinawa City 904-0103, Japan

**Keywords:** diverticular bleeding, HSE, epinephrine, clipping

## Abstract

Epinephrine local injection is a hemostatic procedure used in active diverticular bleeding that elicits vasoconstriction and tamponade effects. We compared the additional benefit of combination therapy with HSE-C (hypertonic saline epinephrine injection with clipping) to clipping monotherapy. Retrospective data on diverticular bleeding between 2011 and 2016 was reviewed. Those with an active bleeding source confirmed by colonoscopy (excluding non-bleeding vessels and adherent clots) who received either HSE-C or clipping were evaluated. Endpoints were rates of successful primary hemostasis, recurrent bleeding, and surgical intervention during hospitalization. A total of 320 patients with diverticular bleeding were evaluated, on which either HSE-C (n = 35) or clipping monotherapy (n = 18) was performed. Rates of successful primary hemostasis (91.4% vs. 66.7%, *p* = 0.048) and direct placement of endoclips (60.0% vs. 16.7%, *p* = 0.004) were significantly higher in the HSE-C group. Although not statistically significant, the HSE-C group had a higher rate of early rebleeding (18.8% vs. 8.3%, *p* = 0.653), while no difference was seen in the number of patients requiring surgery (11.4% vs. 5.5%, *p* = 0.651). HSE-C is associated with a higher rate of successful primary hemostasis for severe active diverticular bleeding but has no significant difference in reducing early recurrent bleeding or the number of patients requiring surgery, suggesting that hemostatic effects may be temporary.

## 1. Introduction

Acute lower gastrointestinal bleeding is seen in approximately 35.7–87.0 patients per 100,000 population annually [1,2], with an incidence rate related to gender and age [3]. The main causes of acute lower gastrointestinal bleeding (LGIB) include diverticulosis, ischemic colitis, polyps, and mucosal abnormalities [4], with diverticular bleeding reported to be the most common cause of bleeding [5]. While spontaneous hemostasis is achieved in around 75–80% of diverticular bleeding [3,6], TAE (trans-arterial embolization), emergency colectomies, and blood transfusions may be required in severe cases. Upon colonoscopy, the source of the bleed is identified in around 22% of patients, for which coloscopy can provide therapeutic intervention as well as a diagnosis [7]. Multiple endoscopic techniques are used to achieve hemostasis such as clipping, injection with diluted epinephrine, contact or non-contact thermal therapy, endoscopic band ligation (EBL), and over-the-scope clip (OTSC) [8,9,10,11,12,13,14]. These can be used in solitude or in combination; however, there is no consensus on the optimal therapy. A systematic review and meta-analysis comparing the effectiveness of each method of hemostasis showed that initial hemostasis and prevention of early recurrent bleeding were equivocal; however, ligation therapy was found to be superior to others in avoiding TAE and surgery [15].

However, EBL has problems due to its technical success being affected by the size and rigidity of the bleeding diverticulum [16] and because the total removal of the scope prior to the procedure is required, even in cases with severe bleeding where urgent action is needed. Meanwhile, local epinephrine (1:10,000) injection is also used to treat diverticular bleeding due to its vasoconstrictive attributes and simple tamponade effect [17,18]. In Japan, injections of epinephrine diluted (1:20,000) with hypertonic saline (HSE) are used as the saline extends the vasoconstriction effect [19]; however, the general consensus is not to use this as monotherapy because the effects are temporary and longstanding hemostasis may not be expected [20]. Colonoscopy with a combination of local epinephrine injection and clipping is reported to be useful in controlling acute diverticular bleeding due to an improved view of the colonic wall prior to clip application [21]. However, previous reports and studies applying this method included only a few cases [9,21,22]. Our aim was to evaluate the efficacy and potential clinical benefit of combination therapy with HSE local injection and clipping (HSE-C) in active diverticular bleeding by comparing clinical outcomes to those in patients undergoing clipping as a monotherapy.

## 2. Materials and Methods

### 2.1. Study Setting

**Figure 1:**
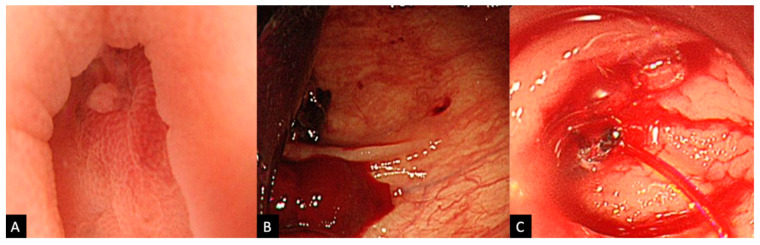
Stigmata of recent hemorrhage for diverticular bleeding. (**A**) A non-bleeding visible vessel within a diverticulum. (**B**) Adherent clot covering the opening of a diverticulum. (**C**) An active bleeding vessel.

We retrospectively reviewed the hospitalization and follow-up data of patients who were admitted with LGIB that was attributed to diverticular bleeding between January 2011 and October 2016 at Urasoe General Hospital. At our unit, in patients who present with relatively severe non-tender hematochezia and low hemoglobin levels, if not contraindicated, contrast computed tomography (CT) is performed to seek extravasation from a diverticulum, after ruling out other conditions that have a similar presentation such as hemorrhoids and ischemic colitis. Clinical diagnosis of diverticular bleeding is made after comprehensive assessment. In patients who are negative for extravasation and are not deemed to have active bleeding, spontaneous hemostasis is judged to have been achieved and elective colonoscopy is performed after full bowel preparation, usually on the following working day. Colonoscopy with preparation is the standardized treatment for diverticular bleeding; however, those with extravasation are judged to be hemodynamically unstable, and either emergency colonoscopy without preparation or TAE is performed. TAE is preferred if vital signs are particularly unstable or if there is a previous record of difficulty in scope insertion; however, for most cases, emergency colonoscopy is the method of choice. The decision of performing a blood transfusion is made after comprehensive consideration including hemoglobin levels, physical examination findings, the severity of the hematochezia, and CT findings, with the restrictive transfusion threshold being set at a hemoglobin level of around 7–8 g/dL, or slightly higher for those with cardiovascular disease. During the study period, HSE-C and clipping monotherapy were the standardized approaches for hemostasis of severe active diverticular bleeding and were selected depending on the endoscopists’ preferences. Exceptional cases treated by alternative treatments such as EBL were excluded. Typically, adherent clots, visible vessels, and active bleeds, which are commonly referred to as stigmata of recent hemorrhage (SRH), are considered the bleeding source (Figure 1), providing a definitive diagnosis of diverticular bleeding as well as an indication for endoscopic treatment [23,24]. Since HSE local injection is aimed at achieving temporary hemostasis, it is unnecessary to inject before attempting to clip adherent clots and visible vessels. Therefore, in principle, HSE-C is only used for visible active bleeds, and data on patients with active diverticular bleeding, excluding adherent clots and visible vessels were evaluated in this study. All unsuccessful cases with persistent active bleeding underwent TAE or surgery, with TAE being attempted in those with an identified bleeding source on CT imaging or colonoscopy and surgery being performed in those with persistent bleeding after failed TAE or if there was persistent bleeding and the source was uncertain.

### 2.2. Procedure

**Figure 2:**
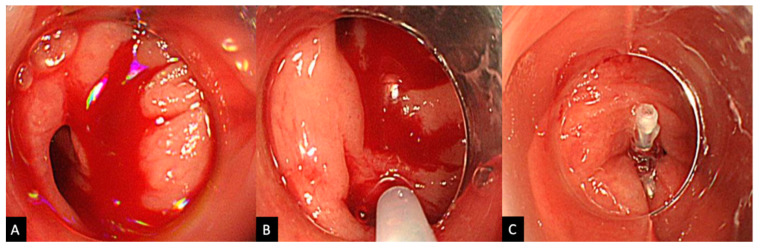
(**A**) Endoscopic view of active bleeding from a diverticulum. (**B**) Local injection of epinephrine administered around the neck of the bleeding diverticulum. (**C**) Primary hemostasis achieved after direct clipping.

**Figure 3:**
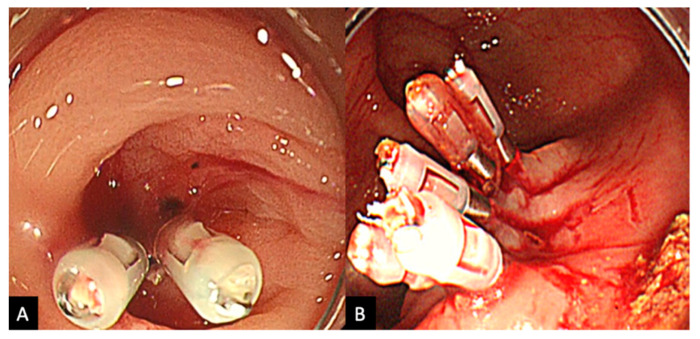
(**A**) Direct clipping: clips are administered directly upon the bleeding diverticulum. (**B**) Indirect clipping: bleeding diverticulum is sealed off indirectly using a zipper-like method.

The procedure was performed by various endoscopists including non-experts; however, all cases were performed under expert supervision to ensure quality of practice. In those undergoing HSE-C, once the source of active bleeding is identified, between 0.5 mL and 2.0 mL of HSE solution is injected into the submucosa around the neck of the responsible diverticulum, a process which is repeated 1–4 times (Figure 2). Injections are generally repeated until the bleeding is weakened enough to gain an improved visual field, so that clipping can be performed in a stable environment. HSE-C injection was abbreviated in the monotherapy group. The clipping method administered was either a direct method, where a clip is placed directly into the diverticulum and onto the bleeding vessel, or an indirect method, where the opening of the bleeding diverticulum is indirectly closed off via a zipper method (Figure 3). Direct clipping is generally the method of choice, with indirect clipping only performed when visibility is insufficient or if the maneuverability of the scope is poor. Colonoscopy was performed using one of the following scopes (PCF-Q260AZI, PCF-H290ZI, PCF-H290I, CF-HQ290I, Olympus Medical Systems, Tokyo, Japan), fitted with a transparent hood, and EZ clips (Olympus Medical Systems) were used for clipping.

### 2.3. Outcomes

Our aim was to demonstrate the efficacy of the combination of local epinephrine injection and clipping; thus, the primary outcome was set as successful primary hemostasis, that is, complete cessation of bleeding to an extent that justifies the total removal of the endoscope, as clearly indicated on the endoscopy procedure report. Secondary outcomes were early recurrent bleeding rates, which we defined as bleeding within 30 days post-procedure, and a requirement for surgery during hospitalization. We also measured mortality, the location of the bleeding, blood transfusion requirements, clipping method (direct vs. indirect), the amount of epinephrine used, and adverse events.

### 2.4. Statistical Analysis

Statistical analysis was performed using EZR (Easy R) version 1.54 (Saitama Medical Center, Jichi Medical University, Saitama, Japan). The Student’s t test and Fisher exact test were applied to continuous and categorical variables, respectively, and a *p* value < 0.05 was considered statistically significant.

## 3. Results

### 3.1. Patient Characteristics

**Figure 4:**
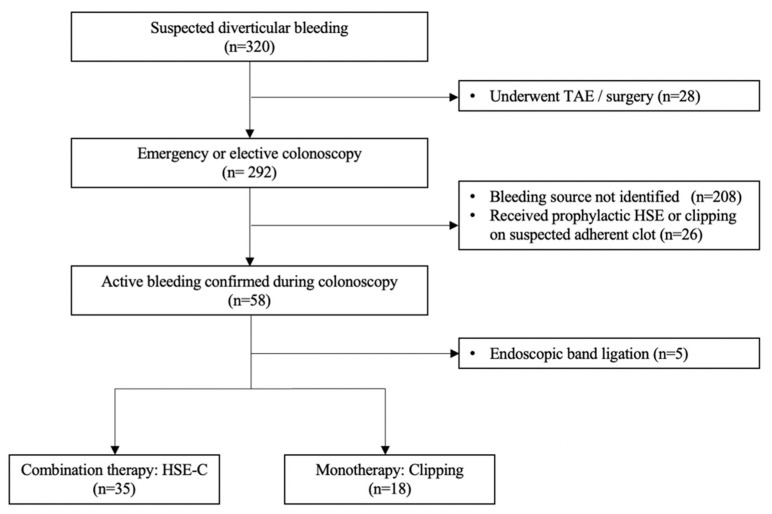
CONSORT diagram of the study.

A total of 320 patients presented with suspected diverticular bleeding between January 2011 and 28 October 2016, all of which underwent emergency TAE or surgery as their initial treatment. Of these, 292 patients received a colonoscopic investigation during their hospitalization, of which 208 were judged to have achieved spontaneous hemostasis; 26 cases had no active bleeding but received prophylaxis with HSE injection or clipping on the suspected vessel or clot. Active bleeding was confirmed in 58 (19.9%) cases, with five patients undergoing EBL; thus, 35 patients who underwent HSE-C and 18 patients who underwent monotherapy with clipping were eligible for the analysis (Figure 4). The baseline characteristics of these patients are listed in Table 1.

The mean age was significantly higher in the HSE-C group (76.7 ± 9.3 years) compared to the clipping group (69.4 ± 9.7 years; *p* = 0.011). Although distributed differently throughout the colon, the rate of right-sided bleeding (cecum, ascending colon, and transverse colon) in the clipping group (88.9%, 16/18) was higher than in the HSE-C group (57.2%, 20/35; *p* = 0.029). The proportion of male patients, concurrent hypertension, diabetes mellitus, history of coronary artery disease, usage of anticoagulant or antiplatelet agents, and extravasation on CT scans were similar between the two groups.

### 3.2. Outcomes

The clinical outcomes of the HSE-C and clipping for the treatment of active diverticular bleeding are shown in Table 2.

Rates of successful primary hemostasis (91.4% vs. 66.7%, *p* = 0.048) and direct placement of endoclips (60.0% vs. 16.7%, *p* = 0.004) were significantly higher in the HSE-C group compared to the clipping group; however, no statistical difference was seen between early recurrent bleeding rates (18.8% vs. 8.3%, *p* = 0.653). All patients with failed primary hemostasis in both groups received emergency TAE. Of the six patients with recurrent bleeding in the HSE-C group, three patients received re-attempted clipping and three received TAE, with four of those going on to receive surgery as a result of persistent bleeding. One patient in the clipping group received surgery during hospitalization, with no significant difference seen between the two groups (11.4% vs. 5.5%, *p* = 0.651). The total amount of epinephrine injected was 2.1 ± 1.5 mL, with the number of injections being 2.5 ± 1.3. Outcomes with regard to the clipping method are summarized in Table 3.

The rate of successful primary hemostasis (direct vs. indirect) was relatively lower when clipping was applied directly in both the HSE-C (85.7% vs. 100%) and clipping (33.3% vs. 73.3%) groups. However, direct clipping scored a lower rate of recurrent bleeding in both groups (HSE-C: 16.7% vs. 21.4%, Clip: 0% vs. 9.1%).

### 3.3. Adverse Events

**Figure 5:**
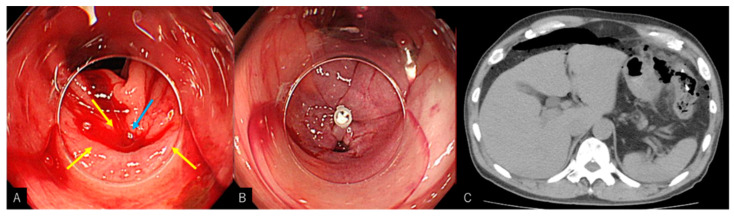
A case of delayed perforation occurred after HSE-C (hypertonic saline epinephrine injection with clipping) at the splenic flexure. (**A**) 1.5 mL HSE was injected at 3 points (yellow arrow), adding up to 4.5 mL, and a single clip was placed indirectly at the neck of the diverticulum (blue arrow). (**B**) Hemostasis was confirmed, and further clipping was not possible due to the instability of the scope. (**C**) CT scans revealed free air in the abdomen on day 5 and subsequent emergency surgery was performed.

A severe adverse event was seen in one patient in the HSE-C group, who had developed delayed colonic perforation. The patient, in his mid-sixties and being treated for hypertension, had initially achieved primary hemostasis in the transverse colon with three injections and a single indirect clip (Figure 5). Five days post-procedure, he displayed signs of pyrexia and abdominal pain, and his diagnosis was made following a CT scan for which emergency surgery was performed. No adverse events were seen in the clipping group. No patient deaths due to diverticular bleeding or its associated complications were reported in either group.

## 4. Discussion

Recent therapeutic advances have given rise to an abundance of hemostatic techniques that endoscopists have at their disposal for the treatment of diverticular bleeding, such as CT-guided percutaneous injections of epinephrine and cyanoacrylate, with various new techniques typically seen in the upper gastrointestinal tract, which may become applicable to diverticular bleeds in the future [25,26,27]. Epinephrine local injection is a simple and basic therapy first reported in the late 1980s for diverticular bleeding with high rates of hemostasis [17,18]. Combination therapy has become a well-established technique despite there being few updates in the existing literature and continues to be recommended without sufficient scientific evidence. To the best of our knowledge, this is the first study to focus on combination therapy with epinephrine injection and clipping, and features the largest sample size of this method. Our study demonstrates the potential benefit of HSE-C in controlling bleeding in the acute stage of severe diverticular bleeding and provides new insight into its efficacy and associated precautions.

HSE-C brought about a higher rate of primary hemostasis in comparison to monotherapy with clipping, which indicates that it contributes to controlling bleeding in the very acute stage and will reduce the need for urgent radiological or surgical intervention. However, given that no significant difference was seen in the rate of early recurrent bleeding or requirements for surgery during hospitalization, the hemostatic effect of HSE-C may be temporary. It can be hypothesized that the temporary vasoconstrictive tamponade effect of HSE may cause misrecognition of unsuccessful clipping, which may result in a recurrent bleeding event. Although reports on monotherapy with local epinephrine injection are limited, it has been reported that primary hemostasis has been achieved in 100% of patients [10,18,28], while primary hemostasis for monotherapy with clipping was reported to be around 96% [20], higher than the rates in our study (HSE-C 91.4%, clipping 66.7%). It should be noted that the rate of active bleeding was considerably low, as adherent clots were included as an indication for endoscopic intervention in prior studies. Limiting analysis to active bleeds in our study was important in order to evaluate the clinical benefits of combination therapy, in that the administration of epinephrine was the most effective therapy in this setting. Although a simple comparison of the data with the existing literature is difficult, by comparing HSE-C and clipping, we were able to show that injection with HSE-C prior to clip application in severe active bleeding brought about a higher rate of primary hemostasis and a higher rate of direct clip placement.

Previous single-center studies comparing the efficacy of direct and indirect clipping were inconsistent and failed to give a definitive conclusion in regard to their clinical efficacy [29,30,31]. However, in a recent large, multi-center cohort study conducted by Kishino et al. [32], direct clipping was found to be independently associated with a reduced risk of early (within 30 days) rebleeding, suggesting that direct clipping should be chosen, despite the rate of successful primary hemostasis being similar between the two methods. However, no significant difference was seen in rebleeding rates in active bleeding, which the authors attributed to the obscurity of the bleeding point prior to application. The feasibility rate of direct clipping for active bleeding was around 20% (27/136) in previous studies [30,33], a figure similar to that in our study for clipping without HSE injection (16.7%). We believe that prior administration of HSE made a contribution to obtaining an improved view of the bleeding site prior to clip administration, allowing direct placement of clips to be possible (60%).

Another reliable endoscopic treatment alternative to endoscopic clipping is EBL. EBL closes off the bleeding diverticulum completely with a high rate of primary hemostasis (93%) [20] and may be utilized in non-diverticular acute lower gastrointestinal bleeds [34]. There are multiple studies that report EBL as a superior treatment [11,35]. However, the size, location, and stiffness of the diverticulum affects the technical success of the procedure [16], and two cases of post-procedural perforation have been reported [36,37]. While both are important therapeutic methods, HSE-C can be applied to any diverticula without requiring withdrawal of the colonoscope. There are no known reports of combination therapy with HSE injection and EBL; hence, this may be considered in the future. The cost of the equipment required for EBL and endoscopic clipping is USD 130/kit and USD 9/clip, respectively, with the use of reusable, reloadable devices [16]. Not only is epinephrine cheap (USD 0.85/mg) and readily available, local injection prior to clipping does not require additional hassle, and can be undergone in one swift series of events.

We recorded one serious adverse event of delayed intestinal perforation in the HSE-C group. Initially, primary hemostasis was achieved using a solitary indirect clip, after a total of 4.5 mL of HSE was injected at three points around the diverticulum, which was relatively higher than the average amount of HSE injected in this study (2.5 mL). Given that perforation occurred 5 days after procedure and only one indirect clip was applied, it is most likely that colonic ischemia brought about by the vasoconstrictive effect of HSE was responsible, rather than injury caused by the clipping. The patient received an emergency transverse colectomy and subsequent colostomy closure 6 months later without any further adverse events. Although HSE-C is a reliable treatment method, care should be taken to minimize the amount of HSE injected, and routine use of epinephrine cannot be recommended for cases without active bleeding or when clipping can be performed under stable, clear vision.

Our study is limited by its retrospective nature and limited sample size. Although largely insignificant, differences in age, bleeding site, and antithrombotic agents were not negligible in the baseline characteristics, hinting at a potential selection bias. Right-sided bleeding is associated with a lower rebleeding rate and, therefore, may have had a favorable effect on the HSE-C group [32]. Given that the identification rate of SRH in colonic diverticular bleeding is low (22%), most prior studies have limited sample sizes [38]. Considering that our study included a larger sample size than prior studies, despite the exclusion of adherent clots and non-bleeding vessels, we believe that our data suggests that HSE injection is associated with a higher rate of successful hemostasis for active diverticular bleeding. It should be noted that there were a few patients who could not visit the hospital due to poor ADL or who were lost to follow up (9.4%, 5/53), although follow-up appointments are booked 2 to 4 weeks post-discharge in most patients. This may have led to an underestimation of rebleeding episodes if these patients had presented at a different hospital. Since there was no concrete protocol and the decision was dependent on the preference of the colonoscopist, there may be a degree of variability in the hemostatic method chosen and the amount of HSE injected. However, in principle, HSE-C is utilized in patients with severe bleeds; therefore, patients with heavy bleeding and an inadequate visual field are more likely to be seen in the HSE-C group. Since all cases included in this study had severe active bleeding, criteria for TAE and surgery were mostly consistent; cases of failed colonoscopic procedures were largely TAE, and cases which required further treatment for recurrent bleeding received surgery without attempts at other types of treatment.

## 5. Conclusions

In conclusion, the combination of epinephrine local injections and clipping brings about a higher rate of primary hemostasis in patients with active diverticular bleeding but is not associated with a lower rate of early recurrent bleeding or a requirement for surgery during hospitalization, suggesting that hemostatic effects may be temporary. Considering the current narrative, although EBL is recommended as first-line treatment for diverticular bleeding, clipping can also be considered due to the simplicity of the procedure. In cases where EBL is difficult, HSE-C has a role in securing visuality by controlling active bleeding and improving the overall quality of clipping. Patients with active bleeding should be carefully monitored regardless of the treatment strategy. A future multicenter study to further evaluate the efficacy and safety of HSE-C is warranted.

## Figures and Tables

**Table 1:** Baseline characteristics and clinical findings of patients assigned HSE-C or clipping for active diverticular bleeding.

	HSE-C (n = 35)	Clipping (n = 18)	*p* Value
Age ± SD (range)	76.7 ± 9.3 (61–93)	69.4 ± 9.7 (50–83)	0.011 *
Male % (n)	62.9 (22)	77.8 (14)	0.358
Concurrent Disease			
Hypertension % (n)	88.6 (31)	66.7 (12)	0.071
Diabetes mellitus % (n)	8.60 (3)	22.2 (4)	0.211
Coronary artery disease % (n)	22.9 (8)	33.3 (6)	0.515
Antithrombotic agents % (n)	65.7 (23)	38.9 (7)	0.083
Aspirin	40 (14)	38.9 (7)	1.000
Clopidogrel	11.4 (4)	22.2 (4)	0.421
Cilostazol	8.6 (3)	5.5 (1)	1.000
Warfarin	22.9 (8)	11.1 (2)	0.464
Extravasation on CT scan % (n)	70.5 (24)	44.4 (8)	0.155
Right-sided bleeding % (n)	57.2 (20)	88.9 (16)	0.029 *

* *p* value of <0.05 was considered statistically significant.

**Table 2:** Clinical outcomes of patients assigned HSE-C or clipping for active diverticular bleeding.

	HSE-C (n = 35)	Clipping (n = 18)	*p* Value
Successful primary hemostasis % (n)	91.4 (32/35)	66.7 (12/18)	0.048 *
Early recurrent bleeding (within 30 days) % (n)	18.8 (6/32)	8.3 (1/12)	0.653
Requirement for surgery during hospitalization % (n)	11.4 (4/35)	5.5 (1/18)	0.651
Persistent bleeding % (n)	75 (3/4)	100 (1/1)	
Delayed perforation % (n)	25 (1/4)	0	
HSE local injection			
Total amount (mL) ± SD	2.1 ± 1.5	N/A	
Number of injections ± SD	2.5 ± 1.25	N/A	
Transfusion % (n)	45.7 (16/35)	72.2 (13/18)	0.085
Adverse Events % (n)	2.9 (1/35)	0 (0/18)	1.000
Follow-up lost within 30 days of discharge	11.4 (4/35)	5.5 (1/18)	0.651

N/A refers to not applicable. * *p* value of <0.05 was considered statistically significant.

**Table 3:** Clinical outcomes of patients according to the clip application method (direct vs. indirect).

	HSE-C (n = 35)	Clipping (n = 18)	*p* Value
Direct clipping: Indirect clipping (direct %)	21:14 (60)	3:15 (16.7)	0.004 *
Rate of successful hemostasis			
Direct clipping % (n)	85.7 (18/21)	33.3 (1/3)	0.099
Indirect clipping % (n)	100 (14/14)	73.3 (11/15)	0.996
Rate of recurrent bleeding			
Direct clipping % (n)	16.7 (3/18)	0 (0/1)	1.000
Indirect clipping % (n)	21.4 (3/14)	9.1 (1/11)	0.604

* *p* value of <0.05 was considered statistically significant.

## Data Availability

The data are not available for public access due to patient privacy concerns but are available from the corresponding author upon reasonable request.
